# Molecular epidemiology of hepatitis B virus infection in Switzerland: a retrospective cohort study

**DOI:** 10.1186/s12879-015-1234-z

**Published:** 2015-10-30

**Authors:** Cédric Hirzel, Gilles Wandeler, Marta Owczarek, Meri Gorgievski-Hrisoho, Jean-Francois Dufour, Nasser Semmo, Samuel Zürcher

**Affiliations:** Department of Infectious Diseases, Bern University Hospital and University of Bern, Bern, Switzerland; Hepatology Unit, Department of Visceral Surgery and Medicine University Hospital Bern, Bern, Switzerland; Institute for Infectious Diseases, University of Bern, Bern, Switzerland

**Keywords:** Hepatitis B virus, Hepatitis D virus, Genotype, Phylogenetic analysis, Switzerland

## Abstract

**Background:**

Chronic hepatitis B virus (HBV) infection affects up to 7 % of the European population. Specific HBV genotypes are associated with rapid progression to end-stage liver disease and sub-optimal interferon treatment responses. Although the geographic distribution of HBV genotypes differs between regions, it has not been studied in Switzerland, which lies at the crossroads of Europe.

**Methods:**

In a retrospective analysis of 465 HBV samples collected between 2002 and 2013, we evaluated the HBV genotype distribution and phylogenetic determinants, as well as the prevalence of serological evidence of hepatitis delta, hepatitis C and HIV infections in Switzerland. Baseline characteristics of patients were compared across their region of origin using Fisher’s exact test and ANOVA, and risk factors for HBeAg positivity were assessed using logistic regression.

**Results:**

The Swiss native population represented 15.7 % of HBV-infected patients living in Switzerland. In the overall population, genotype D was most prevalent (58.3 %), whereas genotype A (58.9 %) was the predominant genotype among the Swiss native population. The prevalence of patients with anti-HDV antibodies was 4.4 %. Patients of Swiss origin were most likely to be HBeAg-positive (38.1 %). HBV genotypes of patients living in Switzerland but sharing the same original region of origin were consistent with their place of birth.

**Conclusions:**

The molecular epidemiology of HBV infection in Switzerland is driven by migration patterns and not by the genotype distribution of the native population. The prevalence of positive anti-HDV antibodies in our cohort was very low.

**Electronic supplementary material:**

The online version of this article (doi:10.1186/s12879-015-1234-z) contains supplementary material, which is available to authorized users.

## Background

Hepatitis B virus (HBV) infection is a global health problem with more than 240 million people chronically infected worldwide and 15 million of them co-infected with Hepatitis D virus (HDV) [1, 2]. Chronic HBV infection is an important cause of liver-related complications including cirrhosis, hepatic failure and hepatocellular carcinoma [3]. Based on the diversity in the complete HBV genomic sequence, 10 HBV genotypes, A to J, and numerous sub-genotypes have been described to date [4, 5].

Specific HBV genotypes have an impact on the clinical course of disease: for instance, genotype C infection is associated with more severe liver damage and faster progression to hepatocellular carcinoma in comparison to genotype B [6]. Furthermore, several studies showed that the different genotypes may vary in their ability to induce chronic hepatitis and in their treatment response to interferon [6]. Of note, HBeAg positive individuals infected with the two most prevalent European genotypes, A and D, show a different response to interferon: Genotype A infected individuals are more likely to have a sustained response to interferon treatment compared to those with genotype D [6, 7].

The geographic distribution of HBV genotypes is well characterized. In Europe the genotypes A (Northern- and Eastern Europe) and D (Mediterranean basin) are predominant, whereas genotypes B and C are most prevalent in Asia, and A, and E in sub-Saharan Africa [4, 8]. We hypothesized that Switzerland, which is located in the heart of Europe, could be the geographical transition zone between the regions where genotype D and A are predominant. The prevalence of Hepatitis B surface antigen (HBsAg) carriers in the Swiss general population is estimated to be 0.3 % [9], but the HBV genotype distribution and the proportion of patients co-infected with HDV is unknown. In this study we assessed the HBV genotype distribution of patients living in Switzerland by analyzing samples of a large reference laboratory in Switzerland and evaluated the association between genotypes and clinical, as well as virological characteristics, including HBeAg-positivity and serological evidence of HDV infection.

## Methods

### Patients

All HBV-infected Swiss residents who had HBV genotyping as part of their regular medical care performed in our reference laboratory between October 2002 and October 2013 were included in our retrospective study. A medical chart review was conducted to collect detailed information on the most important demographic and clinical characteristics. Demographic characteristics included age, sex, and region of origin. The region of origin was coded according to the patient’s nationality. In individuals who immigrated to Switzerland and were naturalized, the region of birth was considered. The study was approved by the local ethics committee of the University of Bern. Patient records were de-identified prior to analysis. In accordance with Swiss law and the Declaration of Helsinki no written informed consent was obtained for this retrospective study.

### HBV genotyping

From 2002 to 2009 the HBV genotype was determined using the INNO-LiPA HBV Genotyping assay (Innogenetics N.V., Ghent, Belgium) according to the manufacturer’s protocol. From 2010 to 2013 HBV genotyping was performed by direct sequencing using primers published by Mallory et al. and Schildgen et al. [10, 11]. For both methods (INNO-LiPA HBV Genotyping assay and direct sequencing) viral DNA was extracted from the patient’s serum or plasma using NucliSENS easyMAG (bioMérieux, Paris, France) according to the manufacturer’s protocol. If genotyping was performed by direct sequencing, a fragment of 941 nucleotides of the viral polymerase/HBsAg was amplified in a primary PCR (pPCR) using the previously described primers HBV_1F and HBV_4R by Mallory et al. [10]. If needed, a nested PCR (nPCR) was performed using the primers TGGATGTGTCTGCGGC (sense primer) and CKTTGACADACTTTCCAATCAATAG (antisense primer) published by Schildgen et al. yielding a PCR product of 622 nucleotides [11].

All PCR products were analyzed by electrophoresis in a 1 % agar gel and then purified using QIAquick PCR Purification Kit (QIAGEN GMBH, Hilden, Germany) according to the manufacturer’s protocol. The purified amplicons were subjected to bidirectional Sanger sequencing using the previously described primer HBV_1F and the antisense primer published by Schildgen et al. [10, 11] for pPCR products and the primers published by Schildgen et al. [11] for nPCR products yielding a sequencing product of 815 nucleotides and 622 nucleotides respectively. Cycle sequencing was performed according to Platt et al. [12]. After purification of the cycle sequencing products by the QIAGEN DyeEx 2.0 Spin Kit (QIAGEN GMBH, Hilden, Germany) the electropherographs were acquired on a genetic analyzer AB-3130 (Life Technologies Europe BV, Nieuwerkerk, Netherlands) and then processed using SeqMan (DNASTAR Inc., Madison, WI, USA). For in silico sequence analysis and genotype determination the open access interpretation tool geno2pheno was used provided by Genafor at http://www.genafor.org/hbv/hbvpredict.php described by Beggel et al. [13]. By performing direct sequencing combined with in silico analysis using geno2pheno additionally to the genotype the subgenotype could be determined while by the INNO-LiPA assay the genotype only was identified.

### Serologic analysis

If performed in our laboratory, samples tested before April 2009 were analyzed by Abbott AxSYM (Abbott Laboratories, Chicago, USA) for Anti-HBe, Anti-HCV and the HIV status. HBeAg was tested using VIDAS (bioMérieux, Paris, France). After April 2009 these serological parameters were tested using ARCHITECT i2000sr (Abbott Laboratories, Chicago, USA). Anti-HDV was tested with ETI-AB-DELTAK-2 (DiaSorin, Saluggia, Italy). All tests were performed according to the manufacturer’s instruction. If no HBeAg test, HIV, HCV or HDV serology was carried out in our institution, we reviewed the charts to identify analyses which were performed elsewhere.

### Phylogenetic analysis

The phylogenetic analysis was performed using the Phylogeny.fr platform [14] with a set of 136 sequences (all samples from 2010 to 2013) from our study of HBV infected patients in Swiss residents [GenBank accession numbers KM524121 to KM524256] and compared to 15 reference strains from GenBank representing all genotypes from A to G. The sequences analysed were all restricted to the same 413 nucleotide stretch of the viral polymerase containing no gaps, deletions or insertions. The alignment was constructed using clustalW2 software provided by the European Bioinformatic Institute at http://www.ebi.ac.uk/Tools/msa/clustalw2/. The phylogenetic tree was calculated by an improved neighbour-joining method implemented in the BioNJ program [15] as part of the Phylogeny.fr platform. Bootstrap analysis was carried out 1000 times for reliability confirmation of the resulting structure. The phylogenetic tree was rooted for non-D genotypes.

### Statistical analysis

In order to analyze the genotype distribution among patients living in Switzerland with different migration background, we classified all patients into one of five “regions of origin”: Switzerland, Europe/Mediterranean, sub-Sahara Africa, Asia and unknown/other. The distribution of regions of origin of our study population was compared with estimates from the general population living in Switzerland, obtained from the federal office of statistics [16]. Baseline characteristics of patients were compared across their original region of origin using Fisher’s exact test and ANOVA for categorical and continuous variables, respectively. The genotype distribution according to region of origin was described using proportional bar charts.

Risk factors for HBeAg-positivity were assessed using logistic regression. The following explanatory variables were included in univariable models: sex, age, region of origin, HBV genotype, HCV-coinfection, HIV-coinfection and serological evidence of HDV-infection. Variables significantly associated with the outcome variable (*p* < 0.05) were included in a multivariable regression model. Multiple imputation was used to impute missing HIV and HCV measurements at baseline, with analyses run on each of 20 datasets and results combined with Rubin’s rules [17]. In a sensitivity analysis, the results of multivariable analyses from the model using multiple imputation were compared to those obtained from complete-case analyses. All analyses were performed using Stata software version 12.0 (College Station, Texas, USA).

## Results

### Baseline characteristics

**Fig. 1:**
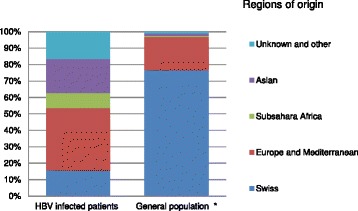
Regions of origin of HBV infected individuals in comparison to the general population in Switzerland. Left bar: distribution of regions of origin of the study population. Right bar: regions of origin of the overall population of Switzerland. *Data from Swiss Federal office for statistics [16]

Between October 2002 and October 2013, the samples of 465 patients living in Switzerland were analyzed. The patients originated from 57 different countries and almost two-thirds of them were men (63.4 %, Table 1). The median age was 37 years (interquartile range (IQR) 29–47 years). Native Swiss individuals represented 15.7 % of the study population, whereas 38 % of patients were from countries in Europe and the Mediterranean basin, 20 % were of Asian and 9 % of sub-Saharan African origin (Fig. 1). This was in strong contrast with the general population of Switzerland, where Swiss natives represented over 80 % of the total number of people [16]. Of note 97.2 % of the Europe and Mediterranean group patients originated from Mediterranean countries (Fig. 1).

**Table 1:** Characteristics by regions of origin

	N	All	Switzerland	Europe and Mediterranean	Sub-Saharan Africa	Asia	Unknown and other	*p* -value
			n(%)	n(%)	n(%)	n(%)	n(%)	
Total	465		73 (15.7 %)	177 (38.1 %)	42 (9.0 %)	96 (20.6 %)	77 (16.6 %)	
*Sex*								0.02
Female	170	36.6 %	19 (35.2 %)	63 (35.6 %)	11 (26.2 %)	46 (47.9 %)	31 (40.3 %)	
Male	295	63.4 %	54 (64.8 %)	114 (64.4 %)	31 (73.8 %)	50 (52.1 %)	46 (59.7 %)	
Median age	465	37	45	37	30	37	37	<0.001
		(IQR 29–47)	(IQR 35–55)	(IQR 29–46)	(IQR 26–36)	(IQR 29–44)	(IQR 28–46)	
*Genotype*	465							<0.001
A	74	15.9 %	43 (58.9 %)	6 (3.4 %)	12 (28.6 %)	4 (4.2 %)	9 (11.7 %)	
AG	1	0.2 %	0 (0.0 %)	0 (0.0 %)	0 (0.0 %)	0 (0.0 %)	1 (1.3 %)	
B	37	8.0 %	0 (0.0 %)	0 (0.0 %)	0 (0.0 %)	32 (33.3 %)	5 (6.5 %)	
C	41	8.8 %	5 (6.9 %)	0 (0.0 %)	0 (0.0 %)	33 (34.4 %)	3 (3.9 %)	
D	271	58.3 %	23 (31.5 %)	170 (96.1 %)	2 (4.8 %)	27 (28.1 %)	49 (63.6 %)	
E	32	6.9 %	0 (0.0 %)	0 (0.0 %)	28 (66.7 %)	0 (0.0 %)	4 (5.2 %)	
F	5	1.1 %	2 (2.7 %)	0 (0.0 %)	0 (0.0 %)	0 (0.0 %)	3 (3.9 %)	
G	4	0.9 %	0 (0.0 %)	1 (0.6 %)	0 (0.0 %)	0 (0.0 %)	3 (3.9 %)	
*HBe Antigen Status*	391		63	161	39	89	39	<0.001
positive	115	29.4 %	24 (38.1 %)	28 (17.4 %)	11 (28.2 %)	39 (33.7 %)	13 (33.3 %)	
negative	276	70.6 %	39 (61.9 %)	133 (82.6 %)	28 (71.8 %)	50 (66.3 %)	26 (66.7 %)	
*HDV Status*	338		53	141	37	77	30	0.03
HDV positive	15	4.4 %	2 (3.8 %)	5 (3.6 %)	6 (16.2 %)	1 (1.3 %)	1 (3.3 %)	
HDV negative	323	95.6 %	51 (96.2 %)	136 (96.4 %)	31 (83.8 %)	76 (98.7 %)	29 (96.7 %)	
*HCV Status*	337		55	140	37	78	27	0.06
HCV positive	8	2.4 %	3 (5.2 %)	1 (0.7 %)	0 (0.0 %)	2 (2.6 %)	2 (7.4 %)	
HCV negative	329	97.6 %	52 (94.8 %)	139 (99.3 %)	37 (100 %)	76 (97.4 %)	25 (92.6 %)	
*HIV Status*	232		31	90	37	55	19	<0.001
HIV positive	15	6.5 %	5 (16.1 %)	0 (0 %)	7 (18.9 %)	1 (1.8 %)	2 (10.5 %)	
HIV negative	217	93.5 %	26 (93.9 %)	90 (100 %)	30 (81.8 %)	54 (98.2 %)	17 (89.5 %)	

*HDV* Hepatitis D virus, *HCV* Hepatitis C virus, *HBeAg* Hepatitis B envelope antigen, *HIV* Human immunodeficieny virus, *IQR* Interquartile range

### HBV genotype distribution

**Fig. 2:**
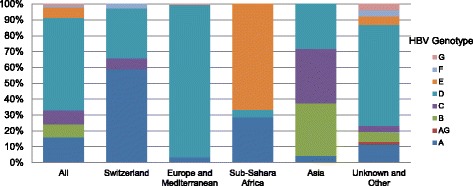
Hepatitis B virus genotypes by region of origin. The bars represent the frequency of the different HBV genotypes by regions of origin

Seven single genotypes could be identified, whereas only one patient had a mixed genotype infection (AG). The genotypes D (58.3 %) and A (15.9 %) predominated in our study population. Among the less prevalent genotypes, C was found in 8.8 % of cases, B in 8.0 % and E in 6.9 %. The HBV genotype distribution varied widely across the regions of origin (Fig. 2). The native Swiss population was mainly infected with genotypes A (58.9 %) and D (31.5 %). Patients originating from other European countries and the Mediterranean basin but living in Switzerland were almost exclusively infected with genotype D (96.6 %). Patients originally coming from sub-Saharan Africa were most commonly infected with genotypes E (66.7 %) and A (28.6 %), whereas genotypes C (34.4 %), B (33.3 %) and D (28.1 %) were most prevalent in those of Asian origin. Importantly, the distribution of HBV genotypes in the group of patients with unknown region of origin was similar to the overall population (Table 1).

### HDV and other co-infections

Hepatitis D serology was available for 72.7 % (338/465) of the study population (Table 1). Anti-HDV antibodies were detected in 4.4 % of the serum samples. Patients originating from sub-Saharan Africa were most likely to be anti-HDV positive (16.2 %), whereas in the Asian group anti-HDV antibodies were least likely to be detected (1.3 %). HCV serology was available for 72.5 % (337/465) of the patients and was positive in 2.4 % of them. The prevalence of HBV/HCV co-infection was very low among the sub-Saharan African and the Europe and Mediterranean groups (0 % and 0.7 %, respectively) but higher in the Swiss group (5.2 %). Only 49.9 % (232/465) of patients had an HIV test result available. Overall, HIV-HBV co-infection was found in 6.5 % (15/232) of the patients and the highest prevalence was observed in patients of sub-Saharan African origin (18.9 %).

### HBeAg positivity

Data on HBeAg status was available for 84.1 % (391/465) of the patients, of which 29.4 % were HBeAg positive. Whereas native Swiss individuals were most likely to have a positive HBeAg (38.1 %), its prevalence was lowest in the European and Mediterranean group (17.4 %). In multivariable analysis, older age was associated with a lower probability of being HBeAg positive (Odds ratio 0.98, 95 % confidence interval 0.96-0.99). In addition, patients of European and Mediterranean origin were less likely to be HBeAg-positive compared to those of Swiss origin (Table 2). There were no statistically significant associations between HBV genotype or HIV-coinfection and HBeAg positivity. Of note, the estimates obtained from the adjusted complete-case analysis were similar to our main results (Additional file 1).

**Table 2:** Risk factors for HBeAg positivity

	N	Odds ratio univariable analysis	*p*-value	Odds ratio multivariable analysis	*p*-value
*Sex*	391				
Female	170	1	0.61		
Male	221	1.12 (0.71–1.78)			
Age	391	0.98 (0.97–0.99)	0.04	0.98 (0.96–0.99)	0.02
*Genotype*	391				
A	67	1		1	
B	31	0.96 (0.38–2.45)	0.93	0.79 (0.23–2.65)	0.70
C	36	2.63 (1.14–6.07)	0.02	1.99 (0.67–5.91)	0.22
D	222	0.77 (0.42–1.42)	0.41	1.57 (0.70–3.54)	0.28
E	28	0.94 (0.36–2.49)	0.90	1.37 (0.35–5.35)	0.64
F	7	3.13 (0.64–15.30)	0.16	4.11 (0.73–23.10)	0.11
*Region of origin*	391				
Switzerland	63	1		1	
Europe and Mediterranean	161	0.34 (0.18–0.66)	0.01	0.24 (0.10–0.57)	0.01
Sub-Saharan Africa	39	0.64 (0.27–1.51)	0.31	0.42 (0.11–1.53)	0.19
Asia	89	1.27 (0.66–2.45)	0.48	1.03 (0.41–2.58)	0.94
Unknown and Other	39	0.81 (0.35–1.88)	0.63	0.53 (0.20–1.40)	0.20
*HIV Status*	228		0.03		0.11
HIV positive	15	4.51 (1.45–14.01)		2.53 (0.82–7.84)	

95 % Conf. Interval in brackets; *HIV* Human Immunodeficiency virus

### Phylogenetics

**Fig. 3:**
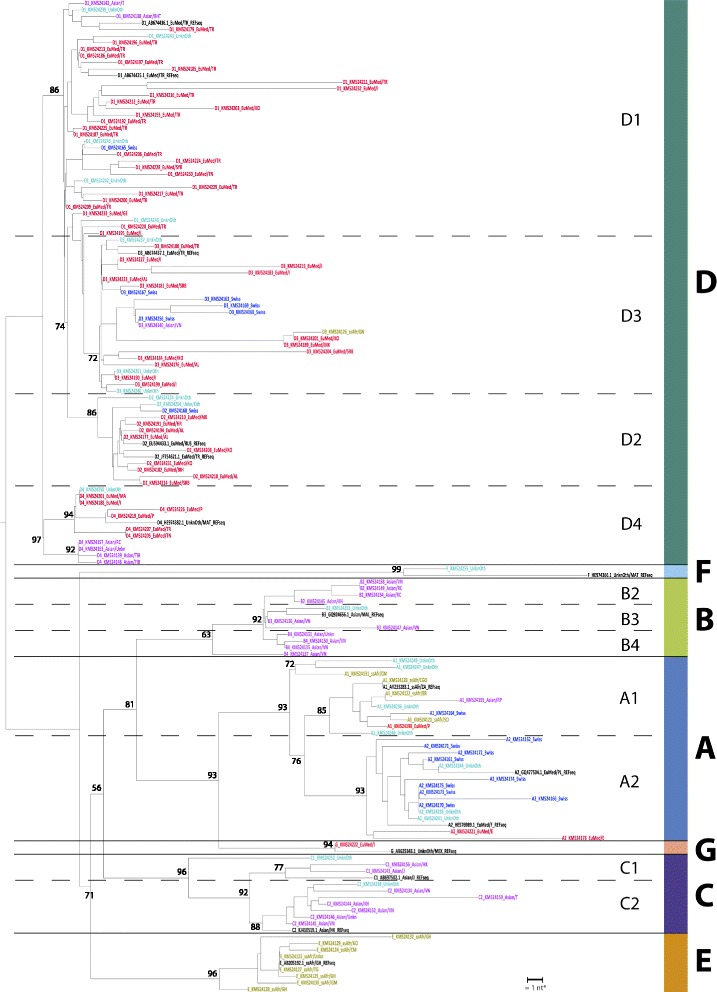
Dendogram of Hepatitis B virus in Swiss residents. Region of origin is illustrated by different colours: Swiss (blue), Asian (purple), European/Mediterranean (EuMed, red), sub Saharan African (ssAfr, green), unknown or other (Unkn/Oth, turquois), reference sequences (REFsequ, black). Each sample is labelled with Genotype_GenBank accession number_region of origin/country code. The country codes are provided in the supplemental material (Additional file 1: Table S2). *The bar length indicates the distance of a 1 nucleotide (nt) polymorphism. The number in the tree indicates the bootstrap reliability

The phylogenetic tree of the partial HBV polymerase gene of our study population is shown in Fig. 3. HBV genotypes D and A were of special interest because they are the predominant genotypes in Europe. The cluster infected with sub-genotype D1 mainly consisted of patients originating from Turkey (belonging to the European and Mediterranean group). Among twenty-two patients of Turkish nationality, twenty were infected with this sub-genotype. With the exception of two samples belonging to patients of unknown origin, the D2 cluster consisted exclusively of patients from the Balkan region (European and Mediterranean group). Of seven patients from the Swiss group carrying HBV genotype D, five were infected with the sub-genotype D3. The D4 cluster consisted of patients originating from China (Asian group) and from the Mediterranean basin. Finally, the A1 cluster consisted predominantly of patients originating from sub-Saharan Africa, whereas the A2 cluster was mainly represented by Swiss native patients.

## Discussion

In this large and representative sample of HBV-infected patients living in Switzerland, we showed that HBV-related epidemiological and virological determinants were driven by migration patterns. The largest group of individuals originally originated from the Mediterranean basin and was predominantly infected with HBV genotype D. We found substantial differences in demographic and virological characteristics between groups of individuals of different regions of origin, including HBV genotype distribution and proportion of viral co-infections. The Swiss native population showed the typical genotype distribution of Northern European countries where patients are most commonly infected with genotype A. Serological evidence of HDV infection was surprisingly low, except for patients of sub-Saharan African origin, of which 16 % were anti-HDV positive. In line with other European studies, older patients were least likely to have a positive HBeAg.

Although only 23.8 % of the population in Switzerland consists of immigrants [16], patients originating from the Mediterranean basin, sub-Saharan Africa as well as Asia represented over 84 % of our study population. The overall HBV genotype distribution showed a typical Mediterranean pattern with a predominance of genotype D mainly due to the large proportion of patients from Portugal, Italy, Turkey and the Balkan region. The genotype distribution among the Swiss native population differed considerably from that of the overall study population and was comparable to patterns described for Germany, Belgium, Poland, the Czech Republic and Scandinavia [8]. As shown previously for Iceland, our study underlines the role played by migration patterns in shaping the epidemiological and virological landscape of HBV infection in countries with a low HBV prevalence in the domestic population [18]. Considering the strong link between HBV genotype and the natural history of disease as well as the better response to interferon treatment in genotype A compared to genotype D infected patients, a careful evaluation of the region of origin as well as the determination of HBV genotype for all patients might be warranted in countries like Switzerland.

Phylogenetic analyses showed that the Swiss native population was mainly infected with the sub-genotypes A2 and D3. Thus, Switzerland seems to be a geographical transition zone for HBV infection, between Italy, where the sub-genotype D3, is most prevalent [19] and northern European countries, where the sub-genotype A2 predominates [8]. The phylogenetic analyses of genotype D strains were particularly informative: Patients of Turkish origin were predominantly infected with the genotype D1, in line with a recent report on HBV infection in Turkey [20]; Furthermore, in our study, HBV D2 virus seemed to circulate primarily among patients who immigrated from the Balkan region which is consistent with the findings of Zehender et al. in Albania [21]. Interestingly four patients of Asian origin (two Tibetans, one Chinese and one of unknown origin) have clustered in subgenotype D4. This is unusual for HBV strains of this region and was not described before except for a report of C/D4 recombinants from Tibet where 96.8 % of the HBV strains analyzed showed a C/D4 recombinant pattern [22]. Due to the short fragment analyzed in our study one cannot exclude that the four samples would belong to the recombinant C/D4 genotype. Taken together, these results show that several homogeneous clusters of HBV-sub-genotypes have established in Switzerland, owing to migration patterns, and that the Swiss native HBV-infected population has a genotype distribution consistent with its geographical situation.

Hepatitis D co-infection is clinically important because it causes more severe liver disease and accelerated progression to cirrhosis leading to higher rates of hepatic decompensation and mortality compared to HBV mono-infection [23]. The prevalence of HBV/HDV co-infection varies widely across European countries, ranging from 2 % in Great Britain to 46 % in eastern Turkey [24, 25]. The overall decreasing prevalence of HDV-co-infection observed in recent years has been attributed to the decline of chronic HBV carriers following the introduction of vaccination programs [2]. In Switzerland, we found 4.4 % of the chronic HBV-infected patients to be anti-HDV positive, which was slightly lower than the 5.9 % estimated in a recent survey completed by physicians treating HBV-infected patients in 2008 [26]. In contrast to the low overall rate of patients with positive anti-HDV antibodies in our study population, 16 % of the patients from sub-Saharan Africa in our study were anti-HDV positive, in line with reported estimates from West Africa [27].

Seventy percent of our study population was HBeAg-negative. The increasing predominance of HBeAg-negative HBV infections was highlighted in a recent French study [28] and a report from Italy showed that the proportion of HBeAg-negative infections increased from 41 % between 1975 and 1985 to 90 % in the 1990′s [29]. In line with other European studies, older patients and those originating from the Mediterranean basin were less likely to have a positive HBeAg [30]. A possible explanation for the significantly lower rate of HBeAg-positive patients among the Europe and Mediterranean group compared to the Swiss group in our study could be the already previously described short duration of the immune reactive HBeAg positive phase in the Mediterranean population with subsequent HBeAg loss in combination with the high prevalence of precore mutations in this population [30].

This is one of the largest studies evaluating epidemiological and virological patterns of HBV infection in a low prevalence country and the first study assessing the HBV genotype distribution as well as the HDV seroprevalence of HBV-infected patients in Switzerland. The detailed virological and phylogenetic analyses allowed us to describe the main determinants of HBV infection in Switzerland, a country with a highly diverse HBV landscape due to its geographical situation as well as its migration patterns. Our study had several limitations. The individual patient data were collected retrospectively, resulting in partially incomplete information on several key variables such as HIV-coinfection. This is of importance as the epidemiological and clinical determinants of HBV infection are linked to the presence of HIV-coinfection [31]. It also underlines the importance of conducting prospective, large cohort studies dedicated to the study of HBV infection. In addition we did not know the region of origin of 70 patients, representing 16.6 % of the study population. However, as the genotype distribution in this group was similar to that of the overall study population, this should not have had a major impact on the overall results. Instead of using full genome phylogenetic analyses (gold standard) to determine HBV sub-genotypes, we analyzed a fragment of the polymerase/HBsAg using the geno2pheno tool. However, to verify the accuracy of our phylogenetic analysis we inserted reference sequences for every HBV subgenotype. Finally, only serological analyses for HDV status were available, therefore the proportion of patients with replicating HDV infection in our cohort was unknown.

## Conclusions

We showed that the molecular epidemiology of HBV infection in low prevalence countries like Switzerland is mainly driven by migration and not by the genotype distribution of the native population. The predominance of genotype D in the overall population can be explained by immigration of patients infected with HBV sub-genotypes D1 and D2 from Turkey and the Balkan region, respectively. The HBV genotype distribution in native Swiss individuals mainly included genotypes A2 and D3, which are most prevalent in neighboring countries. In light of these results and considering the impact of HBV genotypes on the course of disease and treatment response, the region of origin and, ideally genotypes, should be assessed in all patients in order to improve management. To address the most important research questions regarding the impact of HBV genotypes on clinical outcomes and treatment response, large, prospective cohorts of HBV-infected patients need to be established.

## Additional file

Additional file 1: Table S1. Risk factors HBeAg positivity (complete case analysis). **Table S2.** Country codes used for Fig. 3. (DOCX 25 kb)

## Abbreviations

HBV: Hepatitis B virus
HDV: Hepatitis D virus
HBeAg: Hepatitis B envelope antigen
HBsAg: Hepatitis B surface antigen
DNA: Desoxyribonucleic acid
PCR: Polymerase chain reaction
HIV: Human immunodeficiency virus
HCV: Hepatitis C virus
ANOVA: Analysis of variance
NCBI: National center for biotechnology information
