# Supplementary material for: Molecular epidemiology of hepatitis B virus infection in Switzerland: a retrospective cohort study
Source: BMC Infect Dis. 2015 Oct 30;15:483. doi: 10.1186/s12879-015-1234-z (PMC4628344; doi:10.1186/s12879-015-1234-z)
Supplement: Additional file 1: Table S1. — Risk factors HBeAg positivity (complete case analysis). Table S2. Country codes used for Fig. 3. (DOCX 25 kb) [file 12879_2015_1234_MOESM1_ESM.docx]

**Molecular Epidemiology of Hepatitis B Virus Infection in Switzerland: A Retrospective Cohort Study**

Cédric Hirzel ^1^*; e-mail: [cedric.hirzel@insel.ch](mailto:cedric.hirzel@insel.ch)

Gilles Wandeler ^1^; e-mail: [gilles.wandeler@insel.ch](mailto:gilles.wandeler@insel.ch)

Marta Owczarek ^3^; e-mail: [marta.owczarek@ifik.unibe.ch](mailto:marta.owczarek@ifik.unibe.ch)

Meri Gorgievski-Hrisoho ^3^; e-mail: [meri.gorgievski@ifik.unibe.ch](mailto:meri.gorgievski@ifik.unibe.ch)

Jean-Francois Dufour ^2^; e-mail: Jean-Francois.Dufour@insel.ch

Nasser Semmo ^2&^; e-mail: [nasser.semmo@insel.ch](mailto:nasser.semmo@insel.ch)

Samuel Zürcher ^3&^; e-mail: samuel.zuercher@ifik.unibe.ch

1. Department of Infectious Diseases, Bern University Hospital and University of Bern, Switzerland
2. Hepatology Unit, Department of Visceral Surgery and Medicine University Hospital Bern, Bern, Switzerland
3. Institute for Infectious Diseases, University of Bern, Bern, Switzerland

**Table of contents**

1. Supplementary Table 1. Risk factors HBeAg positivity (complete case analysis)
2. Supplementary Table 2. Country codes used for Figure 3

Supplementary Table 1. Risk factors HBeAg positivity (complete case analysis)

|  |  |  |  |  |  |
| --- | --- | --- | --- | --- | --- |
|  |  |  |  |  |  |
|  | N | Odds Ratio Univariable  analysis | p-value | Odds Ratio Multivariable  analysis | p-value |
| ***Sex*** | 391 |  |  |  |  |
| **Female** | 170 | 1 | 0.61 |  |  |
| **Male** | 221 | 1.12 (0.71-1.78) |  |  |  |
| **Age** | 391 | 0.98 (0.97-0.99) | 0.04 | 0.98 (0.96-1.01) | 0.20 |
| ***Genotype*** | 391 |  |  |  |  |
| **A** | 67 | 1 |  | 1 |  |
| **B** | 31 | 0.96 (0.38-2.45) | 0.93 | 1.07 (0.23-4.81) | 0.70 |
| **C** | 36 | 2.63 (1.14-6.07) | 0.02 | 1.97 (0.43-8.95) | 0.38 |
| **D** | 222 | 0.77 (0.42-1.42) | 0.41 | 1.58 (0.52-4.77) | 0.42 |
| **E** | 28 | 0.94 (0.36-2.49) | 0.90 | 1.27 (0.27-5.92) | 0.77 |
| **F** | 7 | 3.13 (0.64-15.30) | 0.16 | 4.82 (0.52-44.19) | 0.16 |
| ***Region of origin*** | 391 |  |  |  |  |
| **Switzerland** | 63 | 1 |  | 1 |  |
| **Europe and Mediterranean** | 161 | 0.34 (0.18-0.66) | 0.01 | 0.19 (0.06-0.61) | 0.01 |
| **Sub-Saharan Africa** | 39 | 0.64 (0.27-1.51) | 0.31 | 0.39 (0.08-1.84) | 0.24 |
| **Asia** | 89 | 1.27 (0.66-2.45) | 0.48 | 0.98 (0.29-3.29) | 0.98 |
| **Unknown and Other** | 39 | 0.81 (0.35-1.88) | 0.63 | 0.40 (0.96-1.68) | 0.21 |
| ***HIV Status*** | 228 |  | 0.03 |  | 0.03 |
| **HIV positive** | 15 | 4.51 (1.45-14.01) |  | 4.32 (1.20-15.60) |  |

95% Conf. Interval in brackets; HIV, Human Immunodeficiency virus

Supplementary Table 2**. Country codes used for Figure 3**

| Country Code | Country |
| --- | --- |
| AL | Albania |
| AO | Angola |
| BHT | Bhutan |
| BIH | Bosnia and Herzegovina |
| CGO | Democratic Republic of the Congo |
| CM | Cameroon |
| E | Spain |
| F | France |
| ER | Eritrea |
| GE | Georgia |
| GH | Ghana |
| GM | Gambia |
| GN  HK | Guinea  Hong-Kong |
| HR | Croatia |
| I | Italy |
| J | Japan4 |
| KH | Cambodia |
| KO  MA | Kosovo  Morocco |
| MAT | Martinique |
| MEX | Mexico |
| MK | Macedonia |
| P | Portugal |
| PL | Poland |
| RC | Republic of China |
| RP | Philippines |
| RU | Russia |
| SRB | Serbia |
| SO | Somalia |
| SYR | Syria |
| T | Thailand |
| TIB | Tibet (belonging to Republic of China) |
| TG | Togo |
| TN | Tunisia |
| TR | Turkey |
| VN | Vietnam |
